# Evaluating the probability of CRISPR‐based gene drive contaminating another species

**DOI:** 10.1111/eva.12939

**Published:** 2020-04-17

**Authors:** Virginie Courtier‐Orgogozo, Antoine Danchin, Pierre‐Henri Gouyon, Christophe Boëte

**Affiliations:** ^1^ Institut Jacques Monod CNRS UMR 7592 Université de Paris Paris France; ^2^ Institut Cochin INSERM U1016 – CNRS UMR8104 – Université Paris Descartes Paris France; ^3^ Institut de Systématique, Évolution, Biodiversité Muséum National d'Histoire Naturelle CNRS Sorbonne Université EPHE UA Paris France; ^4^ ISEM Université de Montpellier CNRS EPHE IRD Montpellier France

**Keywords:** biotechnology, genetically modified organisms, hybridization, molecular evolution, population genetics, risk assessment, wildlife management

## Abstract

The probability D that a given clustered regularly interspaced short palindromic repeats (CRISPR)‐based gene drive element contaminates another, nontarget species can be estimated by the following Drive Risk Assessment Quantitative Estimate (DRAQUE) Equation: $D=\left(hyb+transf\right)\times express\times cut\times flank\times immune\times nonextinct$ with *hyb* = probability of hybridization between the target species and a nontarget species; *transf* = probability of horizontal transfer of a piece of DNA containing the gene drive cassette from the target species to a nontarget species (with no hybridization); *express* = probability that the *Cas9* and guide RNA genes are expressed; *cut* = probability that the CRISPR‐guide RNA recognizes and cuts at a DNA site in the new host; *flank* = probability that the gene drive cassette inserts at the cut site; *immune* = probability that the immune system does not reject *Cas9*‐expressing cells; *nonextinct* = probability of invasion of the drive within the population. We discuss and estimate each of the seven parameters of the equation, with particular emphasis on possible transfers within insects, and between rodents and humans. We conclude from current data that the probability of a gene drive cassette to contaminate another species is not insignificant. We propose strategies to reduce this risk and call for more work on estimating all the parameters of the formula.

**Abbreviations**
CRISPR: clustered regularly interspaced short palindromic repeats
DRAQUE: drive risk assessment quantitative estimate
GM: genetically modified
HGT: horizontal gene transfer
HTT: horizontal transfer of transposable element
TE: transposable element

## 1 INTRODUCTION

Selfish genetic elements are biological entities that favor their own transmission across generations. Examples include transposons that insert copies of themselves at other places in the genome, homing endonuclease genes that copy themselves at targeted genomic sites, segregation distorters that destroy competing chromosomes during meiosis and maternally heritable microorganisms such as *Wolbachia* that favor progeny of infected females (Agren & Clark, 2018). In recent years, researchers have started to develop clustered regularly interspaced short palindromic repeats (CRISPR)‐based gene drives, named here gene drive for short, with the intention to spread synthetic genetic elements into wild populations. Potential applications of gene drives are numerous and include the elimination of mosquitoes to fight malaria, Zika, and other mosquito‐borne diseases, or alternatively the modification of mosquitoes from vector to nonvector so that they no longer transmit human pathogens (Esvelt, Smidler, Catteruccia, & Church, 2014). Applications are not restricted to public health issues and also include agriculture, with for instance the elimination of invasive and pest species such as *Drosophila suzukii* or the suppression of herbicide resistance in weeds (Scott et al., 2018). Potential uses of gene drive are also reaching the field of conservation biology, with the targeting of rats (*Rattus rattus* and *Rattus norvegicus)* in New Zealand (Leitschuh et al., 2018; Rode, Estoup, Bourguet, Courtier‐Orgogozo, & Débarre, 2019). So far, CRISPR‐based gene drives have only been tested in laboratories or in large indoor cages. They have been shown to efficiently boost their own transmission in yeasts (DiCarlo, Chavez, Dietz, Esvelt, & Church, 2015), Drosophila flies (Champer et al., 2017; Gantz & Bier, 2015; KaramiNejadRanjbar et al., 2018), mosquitoes (Gantz et al., 2015; Hammond et al., 2016; Kyrou et al., 2018), the pathogenic fungus *Candida albicans* (Shapiro et al., 2018) and mice (Grunwald et al., 2019).

A CRISPR gene drive cassette is a piece of DNA that comprises several elements: (a) a gene encoding a guide RNA (gRNA) that can recognize a specific target DNA sequence, (b) a *Cas9* gene encoding a Cas9 endonuclease that can cut DNA at the site specified by the gRNA, (c) sequences at the extremities that are homologous to sequences flanking the target site, so that the gene drive cassette can copy itself at the cleavage site via homology‐directed repair, and (d) optional sequences, for example conferring a trait of interest such as malaria resistance (Esvelt et al., 2014). By converting heterozygotes for the gene drive allele into homozygotes, the gene drive cassette alters Mendelian transmission and can thus spread into wild populations. The release in the wild of a few individuals carrying gene drive constructs is thus expected to be sufficient to transform an entire population after a dozen generations (Deredec, Burt, & Godfray, 2008). Gene drives can be designed to introduce a phenotype of interest in a targeted population either through the introduction of a new gene, or by the inactivation of an endogenous gene via the insertion of the gene drive cassette into it (Esvelt et al., 2014). With introduced genetic changes that decrease viability or fertility, a gene drive can be used to eradicate a targeted population or to reduce its size, while with other types of genetic changes, it is possible to alter the characteristics of a population.

The possibilities offered to humanity in terms of benefits by the new molecular techniques of genome edition CRISPR‐Cas are innumerable but also associated with risks which should be carefully monitored (Zhang, 2019). One obvious risk associated with gene drive is that the sequence may escape from the target species and spread into other species. Such spillover could have devastating effects, such as the extinction of a species, or the modification of a large number of individuals, with potentially important ecological consequences. Compared to natural bacterial CRISPR systems, gene drive cassettes are more compact and contain eukaryotic cis‐regulatory elements, so that they are one step closer to potential contamination of nontarget eukaryote species. The risk of gene drives contaminating another species has been mentioned by several authors (Benedict et al., 2008; Esvelt et al., 2014; National Academies of Sciences & Medicine, 2016; Rode et al., 2019; Webber, Raghu, & Edwards, 2015), but to our knowledge, it has not been examined in detail. Risk assessment studies classically present the value of a risk as a product of two terms: Risk = Probability of occurrence × Damage in case of occurrence. The aim of this paper is to derive a formula to evaluate the probability of occurrence of a drive sequence escaping from the target species and contaminating another species. The damage resulting from such an unwanted event will obviously depend on the concerned species. If it is simply another mosquito, the damage might be limited or could even be seen as a serendipitous positive externality. If it were a keystone species or humans for instance, damages could be very important and hard, not to say impossible to mitigate. This paper does not address these questions but concentrates on the probability that a drive escapes from the target species and contaminates another species. Such an outcome results from a succession of events, and the probability is thus the product of the corresponding conditional probabilities. Certain of these probabilities are still poorly known so that the estimates provided here are very rough. However, producing this formula can have two important effects. First, it can help developers of gene drive technology to contemplate the risk associated with their action and consider its potential magnitude. Second, it can trigger further research for better assessment of these probabilities. A famous example of such an approach is the Drake Equation which aimed at estimating the number of active, communicative extraterrestrial civilizations in the Milky Way galaxy (Burchell, 2006). The equation was written in 1961 by Frank Drake, mainly as a way to stimulate scientific dialogue. A weakness of the Drake equation is that some factors are poorly known. However, it certainly promoted numerous research studies and thoughts among scientists.

Here, we examine CRISPR‐based gene drives and we review the different parameters to be considered to evaluate the risk of transfer to another species. We focus on gene drives that can spread autonomously and we do not consider here binary systems, whose genetic elements are located on different chromosomes. Such binary systems have been proposed as a solution to prevent contamination of nontarget populations. However, they are expected to spread less efficiently than autonomous gene drives (Akbari et al., 2015; Esvelt et al., 2014).

## 2 RESULTS

**FIGURE 1:**
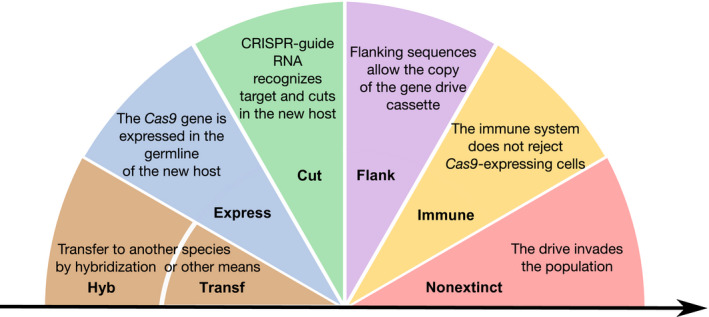
Summary of the different events whose probability must be estimated to assess the risk of gene drive contaminating another species

For a given gene drive construct to contaminate another species, six consecutive steps are required (Figure 1). The probability of contamination can be estimated by multiplying the probability of occurrence of each event, as defined with the following Drive Risk Assessment Quantitative Estimate (DRAQUE) formula:

$$D=\left(hyb+transf\right)\times express\times cut\times flank\times immune\times nonextinct.$$

The bulk probability *D* of the gene drive to contaminate a nontarget species includes (a) the probability that the given piece of DNA passes from the target species to the nontarget species. This can occur by hybridization (*hyb*) or by other means (*transf*). Then, (b) the *Cas9* gene and the guide RNA gene have to be expressed in the new host, with probability *express*. A target sequence must then be recognized and cut by CRISPR‐Cas9 (c) with probability *cut*. The gene drive cassette should insert at the cut site (d) with probability *flank*; the immune system (e) must not eliminate it, with probability *immune,* and finally, it must not be eliminated by stochastic or selective processes (f) with probability *nonextinct*.

This formula could be estimated for one given nontarget species. However, for ease of estimation and practical purposes, we think that it is more relevant to directly define D as the probability that at least one nontarget species is contaminated. We examine below each term of the DRAQUE formula.

### 2.1 Probability of hybridization between the target species and a nontarget species (*hyb*)

The probability that individuals of the target species hybridize with a nontarget species and produce fertile progeny has to be evaluated for each target species, as it may vary among species. We treat here two taxa for which gene drive technology is most advanced, *Drosophila* flies and *Anopheles* mosquitoes.

*Drosophila suzukii* is an invasive pest species originating from Southeast Asia that invaded both America and Europe since 2008 and that attacks ripe fruits (Scott et al., 2018). This species is one of the most advanced systems for potential gene drive applications (Scott et al., 2018). Two closely related species have been described, *Drosophila pulchrella* and *Drosophila subpulchrella*; they are found in Japan, China, India, and Nepal ([Takamori, Watabe, Fuyama, Zhang, & Aotsuka, 2006] and references therein). Recent genome data suggest that hybridization probably occurred recently between *D. suzukii* and *D. subpulchrella*, which diverged about 1–9 million years ago (Conner et al., 2017). Furthermore, fertile hybrids between *D. suzukii* and *D. subpulchrella* have been obtained in the laboratory ([Fuyama, 1983], note that *D. subpulchrella* was erroneously named *D. pulchrella* in the 1983 paper [Muto, Kamimura, Tanaka, & Takahashi, 2018]). Because hybridization between closely related species usually produces hybrids with reduced fitness, it can lead to reinforcement, that is, the increase of reproductive isolation as closely related species diverge (Turelli, Lipkowitz, & Brandvain, 2014). Given that reinforcement is prevalent in Drosophila, one might reasonably speculate that hybridization is common in the wild. Current data thus suggest that gene drives targeting *D. suzukii* may end up in *D. subpulchrella*/*D. pulchrella* if a gene drive ever reaches areas of contact on the Asian continent.

To control malaria with gene drive, two major strategies are currently being developed. One relies on the reduction or suppression of the population of vectors and the other on genetically modifying populations of wild vectors so that they no longer transmit pathogens. The most technically advanced approach is the one conducted by the Consortium Target Malaria aiming at reducing the population of several mosquito species of the *Anopheles gambiae* complex. This complex consists of at least 8 species of the *Anopheles* genus, morphologically indistinguishable and present in sub‐Saharan Africa ([Coetzee et al., 2013] and references therein). Some have a large afro‐tropical distribution (*An. gambiae s.s.*) while others are restricted to savannah area (*An. arabiensis*) or coastal regions (*An. merus* and *An. melas*). It also includes the species *An. quadriannulatus* that is not considered as a malaria vector. The work by Target Malaria is conducted on 3 species of this complex: *Anopheles gambiae s.s.*, *Anopheles coluzzii,* and *Anopheles arabiensis* (https://targetmalaria.org/our‐work/). *An. coluzzii*, formerly known as *An. gambiae* M molecular form, is defined as a separate species since 2013 (Coetzee et al., 2013). Among the *Anopheles gambiae* complex, the question of hybridization has been a subject of interest for geneticists and public health practitioners for decades (Fontaine et al., 2015). Hybridization is of high concern and interest in these mosquito populations due to the potential spread of insecticide resistance between species, and now, with the development of gene drives, of laboratory‐made transgenes.

A classical example of transfer is the geographic expansion and adaptation to arid environment by *An. gambiae* that is associated with an introgression from *An. arabiensis* into *An. gambiae*, resulting from past hybridization between the two species (Besansky et al., 2003; Sharakhov et al., 2006). The presence of the *kdr* resistance (a mutation conferring resistance against pyrethroids, insecticides largely used in impregnated bednets) in the S form of *An. gambiae* (now *An. gambiae s.s.*) and then later in the M form (now called *An. coluzzi*) has also been explained by an introgression rather than by an independent, novel mutation (Weill et al., 2000). This highlights the existence of gene flow between these two species. Recent studies have also highlighted high frequency of hybridization between *An. coluzzi* and *An. gambiae* in West Africa (Caputo et al., 2011; Marsden et al., 2011; Oliveira et al., 2008) and an asymmetric introgression from *An. coluzzi* to *An. gambiae* (Mancini et al., 2015). Genetic exchanges have also been detected between *An. gambiae* s.s. and *An. arabiensis* and led to the idea of particular genomic regions being more prone to cross species boundaries than others (Crawford et al., 2015). In the laboratory, introgression of a synthetic sex ratio distortion system has even been possible from *An. gambiae* to its sibling species *An. arabiensis* (Bernardini, Kriezis, Galizi, Nolan, & Crisanti, 2019). In summary, multiple species inside the *An. gambiae* complex appear to cross‐hybridize, and developers of gene drive technology may aim to develop a system able to target several of them. The higher the number of species, and thus individuals, harboring gene drive constructs, the higher the probability that the gene drive contaminates other species.

Hybridization is common in plants (Goulet, Roda, & Hopkins, 2017; Whitney, Ahern, Campbell, Albert, & King, 2010). Therefore, since the use of genetically modified (GM) plants, the risk of transgene transfer to other species via hybridization has been a general concern (Rizwan et al., 2019; Ryffel, 2014). Unfortunately, despite extensive discussions about this risk, natural populations have rarely been monitored by researchers to search for such transgene escapes from GM fields. Unintended transgene transfers to other species have been demonstrated in rice, canola, sugar beet, soybean, cotton and bentgrass (Table 1). With the diminishing costs of DNA sequencing it is now possible to quantify more broadly the extent of transgene escape events, but whether the necessary funds will be engaged for such investigations is unclear. Conditions have been proposed to decrease the risk of gene transfer, including change in flower color or flowering date (Rizwan et al., 2019; Ryffel, 2014). However, no system totally preventing gene exchange has yet been proposed.

**TABLE 1:** Reported cases of transgene escape from different crops

Crop	From	To	Reference
Bentgrass	*Agrostis stolonifera*	*Polypogon monspeliensis*	Zapiola and Mallory‐Smith (2012)
Canola	*Brassica napus*	*Brassica rapa*	Yoshimura, Beckie, and Matsuo (2006), Warwick, Legere, Simard, and James (2008), Londo, Bautista, Sagers, Lee, and Watrud (2010), Aono et al. (2011)
		*Brassica juncea*	Zhang et al. (2018)
		*Brassica carinata*	Séguin‐Swartz et al. (2013)
Cotton	*Gossypium hirsutum*	*Gossypium barbadense*	Van Deynze, Hutmacher, and Bradford (2011)
Rice	*Oryza sativa*	weedy rice	Chun et al. (2011)
		*Oryza rufipogon*	Wang et al. (2006)
		*Oryza sativa* f. *spontanea*	Serrat et al. (2013)
Soybean	*Glycine max*	*Glycine soja*	Mizuguti et al. (2010)
Sugar beet	*Beta vulgaris* ssp. *vulgaris*	*Beta vulgaris* ssp. *maritima*)	Saeglitz, Pohl, and Bartsch (2000)

### 2.2 Probability of horizontal transfer of a piece of DNA containing the gene drive cassette from the target species to a nontarget species with no hybridization (*transf*)

DNA can be naturally transferred from one eukaryote species to another via so‐called horizontal transfer (HT), through unknown means that may involve vectors such as viruses, microsporidia, mites or parasitoids (Gilbert et al., 2014; Gilbert, Schaack, Pace, Brindley, & Feschotte, 2010; Houck, Clark, Peterson, & Kidwell, 1991; Parisot et al., 2014). In particular, viruses can carry over nucleic acid loads that do not directly belong to the viral‐specific genetic setup, but from the virus host (Gasmi et al., 2015; Gilbert & Cordaux, 2017). Bacteria probably also constitute an important vector to transfer DNA material from one Eukarya species to another. Horizontal gene transfer (HGT) from Bacteria to Eukarya is frequent (Lacroix & Citovsky, 2016), and from Bacteria to Bacteria, it is the norm. Bacteria release their DNA in the environment because they keep lysing either under the action of bacteriophages or during the sporulation process (which entails lysis of the mother cell). This implies that fragments of DNA in the environment or in bacteria can be rapidly contaminated by any novel construct. As cases in point, apparition of antibiotic resistance spreads rapidly from locations where it has first appeared (see, e.g., [Schultz et al., 2017]). HGT from Eukarya to Bacteria has been illustrated in some cases, with viruses as intermediates (Bordenstein & Bordenstein, 2016). Eukaryotic genes in bacteria (EUGENs) are frequent in intracellular parasitic or symbiotic bacteria (e.g., [Hilbi, Hoffmann, & Harrison, 2011]); they may play an efficient role in gene exchange. The DNA pieces that have been observed to undergo HT are usually between 1 kb and several dozens of kb (see HTT‐DB database [Dotto et al., 2015]), which is comparable to the size of gene drive constructs, and can go up to 150 kb in plants and animals (Dunning et al., 2019; Inoue et al., 2017).

Two types of HT can be distinguished, horizontal transfer of transposable elements (HTT) and HGT. Although transposable elements (TEs) also carry genes, this distinction reflects the fact that we know many cases of HTT and relatively few cases of HGT. Most HGT events that occurred in the past between distantly related species are not expected to be detected through comparison of present‐day genomes because most newly inserted DNA sequences are likely to be lost by genetic drift or by selection against the insertion if it is deleterious (i.e., the probability *nonextinct* is zero). The breadth of HGT that can be approached based on comparative studies of actual genome sequences is thus largely underestimated. As a matter of fact, identified HGT events involve pieces of DNA that appear to increase host fitness (HGT, e.g., carotenoid synthesis genes from fungi to pea aphids [Moran & Jarvik, 2010]). Compared to other DNA sequences, TEs have particular characteristics that allow them to integrate into DNA more frequently, and they can also self‐replicate in the new host after HT, so that they are more likely to be noticed. Whether the higher rate observed for HTT than for HGT is only due to the integration and replicative properties of TE is unknown. If viruses are important vectors for HT, the propensity of TE to jump from eukaryote genomes to viruses, and reversely, more frequently than random pieces of DNA (Gilbert et al., 2016) may also explain their higher rate of HT. Compared to TE, a gene drive cassette can also insert itself into a host genome, but its integration into DNA may be less likely and its number of copies in a genome should be lower, so that gene drives may transfer horizontally between genomes less frequently than TEs. However, if TEs are present in the vicinity of the gene drive cassette in the target species, they could end up facilitating the transfer and integration of the DNA in another host via a hitchhiking process. To limit this risk, gene drives should be designed to target genomic regions that are devoid of TEs, if such regions exist in the target species.

Better than TEs, laboratory‐made gene drive constructs resemble homing endonuclease genes, which are naturally occurring mobile elements that bias their inheritance by cutting and inserting themselves at targeted sites within genomes (Agren & Clark, 2018; Burt & Koufopanou, 2004). So far, homing endonucleases have only been found in unicellular organisms and in eukaryotes organelles. Compared to bacterial restriction enzymes, they recognize a long sequence motif, whose size (14–44 bp) is comparable to the one of CRISPR/Cas9 target sites (Hafez & Hausner, 2012). A homing endonuclease gene that specifically targets the *cox1* mitochondrial gene has been transferred independently 70 times between 162 plant species within 45 different families (Sanchez‐Puerta, Cho, Mower, Alverson, & Palmer, 2008). This element is also present in several species of fungi, green algae and liverworts, highlighting extensive HT (Cho, Qiu, Kuhlman, & Palmer, 1998). Unfortunately, no estimate of HT rates for homing endonuclease genes is available.

Cases of HTT have been detected between extremely distantly related species (Gilbert & Feschotte, 2018). For example, the *BovB* element moved at least 11 times between snakes, lizards, ruminants and marsupials (Ivancevic, Kortschak, Bertozzi, & Adelson, 2017) and the *Mariner* element moved between nematodes, arthropods, fungi, molluscs, vertebrates and plants ([Palazzo et al., 2019] and references therein). Numerous stable introductions of virus DNA into the germline of various eukaryote species have also been reported (Chen, Wu, Zhang, Jiang, & Chen, 2016; Feschotte & Gilbert, 2012; Holmes, 2011; Katzourakis & Gifford, 2010). In vertebrates, endogenous retroviruses (ERVs) can insert into their host genome and generate copies of themselves through germline reinfections or retrotransposition events (Gilbert & Feschotte, 2018; Greenwood, Ishida, O’Brien, Roca, & Eiden, 2018). A comparison of ERV sequences in 65 genomes identified no less than 1,000 HT events between distantly related species of vertebrates (Hayward, Cornwallis, & Jern, 2015). Pan‐phylogenomic analyses of ERV sequences revealed that rodents are a major source of retroviruses which they can transmit to other mammals such as livestock (Cui, Tachedjian, & Wang, 2015).

Horizontal transfer events are also ongoing now. The best known case is the worldwide invasion of *D. melanogaster* populations by a TE (*P*‐element) originally present in the distantly related species *D. willistoni* (Clark & Kidwell, 1997; Daniels, Peterson, Strausbaugh, Kidwell, & Chovnick, 1990). This invasion has been carefully recorded and occurred within a few decades during the second half of the last century. Nowadays, the *P*‐element is invading two other Drosophila fly species worldwide, *D. simulans*, originating from *D. melanogaster* (Hill, Schlötterer, & Betancourt, 2016) and *D. yakuba* (Serrato‐Capuchina et al., 2018). *D. simulans* flies sampled before 2010 do not carry the *P*‐element, indicating that this invasion is very recent. The *P*‐element arose in *D. simulans* most likely through hybridization with *D. melanogaster*, but could also have occurred via the unintended escape of a few laboratory‐raised *D. simulans* flies genetically engineered to carry the *D. melanogaster P*‐element. In Mammals, the ERV sequence KoRV‐A is currently observed to invade natural populations of koala *(Phascolarctos cinereus*) (Xu & Eiden, 2015) and herpes virus DNA has started to integrate into human chromosomes (Morissette & Flamand, 2010).

Rough estimates of HTT rates have been obtained recently. Systematic surveys of TEs in complete genomes have inferred HTT events that essentially occurred during the last 10 My. They counted more than 2000 HTT events in 195 insect species (Peccoud, Loiseau, Cordaux, & Gilbert, 2017) and more than 330 HTT in 460 arthropod species (Reiss et al., 2019). (El Baidouri et al., 2014) estimated that more than 2 million HTT occurred between plant species. A study of three *Drosophila* species estimated an average rate of 0.035 HTT per TE family per million years between these three species, with LTR RTs and DNA transposons displaying a higher rate of 0.046 (Bartolomé, Bello, & Maside, 2009). Note that the power to identify HTT events decreases as the HTT events approach the time of species divergence, so that existing quantifications are conservative.

In order to obtain an estimate of the number of contacts a species can have with other eukaryotic species, we used data from the all‐taxa biodiversity inventory (ATBI) conducted in the Great Smoky Mountains National Park (Nichols & Langdon, 2007). As it hosts more than 450 vertebrate species and >8,000 insect species (Discover Life in America website, accessed January 2020 https://dlia.org/smokies‐species‐tally), we can assume that a given species can be in contact with approximately 1,000 distinct Eukaryote species, either directly or indirectly via viruses, bacteria or other microorganisms. As a result, the rate of HTT can be approximated to a minimum of 0.035 transfer events to at least one species per thousand of years (1,000 species × 0.035 HTT events per million years—see paragraph above). This estimate is conservative as it does not take into account horizontally transferred DNA that have disappeared from the recipient genome since their transfer nor HTT events that occurred right after reproductive isolation of the two lineages of interest. In addition, due to the broad geographic distribution of viruses and bacteria that can act as vectors, the probability of transfer to any species among the total estimated 10 millions in the world is probably much higher than this figure. Furthermore, there is no reason to believe that the rate of HTT is constant across time. It is possible that the number of HTT events increases under certain conditions, such as ecological stress or pervasive pathogen infections (Demanèche et al., 2001; Horváth, Merenciano, & González, 2017).

The risk of transfer to nontarget species also depends on the persistence time of gene drives. For replacement/rescue drives, the drive is expected to reach all individuals of the targeted population. Then, in the long term, since there is no selective pressure to maintain a functional endonuclease, the CRISPR‐cas9 cassette can eventually accumulate mutations and thus lose its self‐replicating activity and should eventually disappear from the target population. However, the persistence time of the gene drive cassette should still be relatively long, of the order of several thousands of generations, leaving time at the human scale for possible HGT. The long‐term presence of rescue/replacement drive constructs in the population increases the odds of transfer to nontarget species. As far as we know, no strategy has been proposed to completely remove the gene drive cassette once a population has been fully targeted by a replacement drive.

For eradication/suppression drives, the target population is expected to go extinct, so that no gene drive construct is expected to remain in living organisms. Nevertheless, DNA is a very stable molecule and DNA from dead organisms can make up reservoirs of gene drive constructs. Just taking into account viruses, aquatic ecosystems typically contain 10^6^–10^8^ virus‐like particles per mL (Cunningham, Brum, Schwenck, Sullivan, & John, 2015) and sediments 10^8^–10^9^ particles (Filippini & Middelboe, 2007). DNA is found in all kinds of environments (see [Hunter, Ferrante, Meigs‐Friend, & Ulmer, 2019] for techniques allowing recovery of 1–500 ng of DNA per microliter of water). Some organisms, such as *Acinetobacter baylyi* are able to take it spontaneously into their genome (Mantilla‐Calderon et al., 2019), further amplifying and propagating DNA sequences. Gram‐negative bacteria are cases in point as it has been shown that they rapidly transfer antibiotic resistance to a large number of recipients (Oliveira & Reygaert, 2019).

In summary, recent data indicate that DNA can transfer extensively between distantly related species in all taxon groups. Current quantifications of the probability of transfer of a particular DNA piece to another species are rare and underestimated, and provide a probability of a minimum of 0.035 transfer events to at least one species among 1,000 per thousand years. Whereas the probability of hybridization discussed above is relatively high and concerns a small number of species, the probability of HT discussed here is relatively low but involves a larger number of potential nontarget species. These nontarget species can be very distant both phylogenetically and geographically, due to vectors which can be ubiquitous.

### 2.3 Probability that the guide RNA and *Cas9* genes are expressed in the new host (*express*)

**FIGURE 2:**
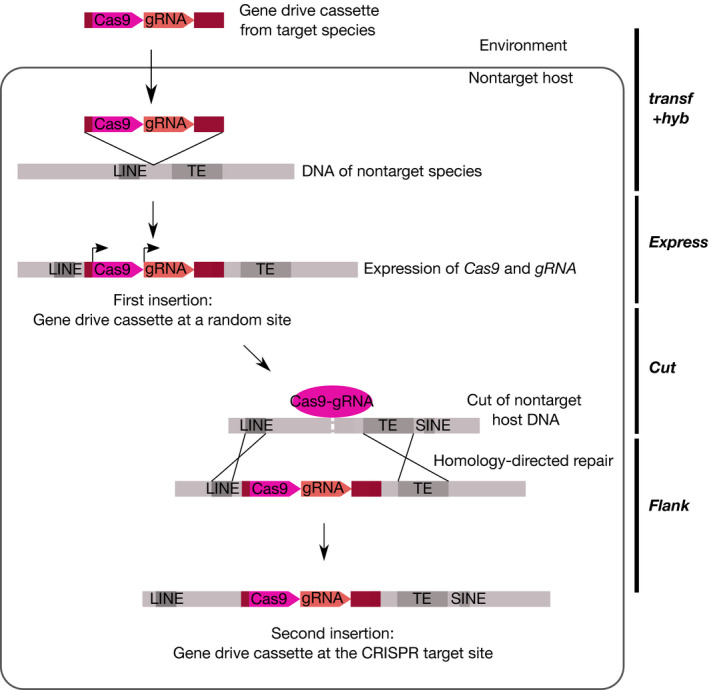
Succession of putative events leading to the insertion of a functional gene drive cassette in another distantly related nontarget species (in case of horizontal transfer with no hybridization). DNA of the nontarget species is indicated in gray and DNA from the target species in warm colors: pink: *Cas9* gene, orange: gRNA gene and brown: neighboring sequences. On the right are different parameters of the DRAQUE equation. In the represented scenario, one LINE (Long Interspersed Element) and one transposable element (TE) serve as flanking sequences for homology‐directed repair. Note that other sequences present in the nontarget host may also be used

For the CRISPR‐Cas9 gene drive system to be active in the nontargeted species, the guide RNA gene and the *Cas9* gene must be expressed in the newly formed zygote or in the new host germline (Figure 2). In other words, potent enhancer regions should be present in the vicinity of the two genes, and promoters should be active to drive expression in the new host (Wittkopp & Kalay, 2012). Experiments swapping enhancers between species suggest that rodent sequences are active across Mammals, while fly/mosquito sequences function across Diptera and sometimes throughout insects. Human enhancers generally drive similar expression in mice (88 My divergence—all divergence times are from TimeTree [Kumar, Stecher, Suleski, & Hedges, 2017]) (Cheng et al., 2014). Enhancers have also been observed to drive expression in corresponding homologous organs in more distantly related species such as mice and bats (94 My divergence [Booker et al., 2016; Cretekos et al., 2008]) or even fish species and humans (465 My divergence [Yuan et al., 2018]). A particular enhancer was shown to drive comparable expression across 9 species of vertebrates including fishes, lizards, snakes and mice (Kvon et al., 2016). In insects, several enhancers have been tested and observed to drive similar expression patterns in *Drosophila*, mosquitoes (248 My divergence) and *Tribolium* (309 My divergence [Cande, Goltsev, & Levine, 2009; Lai et al., 2018]). Several native promoters of Drosophila have been tested and they have been found to function in Lepidoptera (286 My divergence [Imamura et al., 2003; Ramos, Kamal, Wimmer, Cartwright, & Monteiro, 2006; Tamura et al., 2000]) but not in *Tribolium* (Schinko et al., 2010). Native mosquito enhancers are thus unlikely to function in humans (790 My divergence) or in the malaria parasite (1552 My divergence).

To activate expression, published gene drive constructs harbor endogenous cis‐regulatory sequences of germline‐specific genes for the *Cas9* gene and of constitutively expressed genes for the guide RNA genes (Table 2). Therefore, present‐day constructs designed for flies or mosquitoes can be expected to drive expression in the germline across Diptera/insects, and for mice across Mammals.

**TABLE 2:** List of cis‐regulatory sequences used in published gene drive constructs

Target Species	Cis‐regulatory sequences for Cas9 expression	Cis‐regulatory sequences for guide RNA expression	Reference
*Drosophila melanogaster*	*D. melanogaster vasa*	*D. melanogaster U6:3*	Gantz and Bier (2015)
*Anopheles stephensi*	*A. stephensi vasa*	*A. stephensi U6A*	Gantz et al. (2015)
*Anopheles gambiae*	*A. gambiae vasa2*	*A. gambiae U6*	Hammond et al. (2016)
*Drosophila melanogaster*	*D. melanogaster vasa* *D. melanogaster nanos*	*D. melanogaster U6:3*	Champer et al. (2017)
*Drosophila melanogaster*	*D. melanogaster Sry‐alpha* *D. melanogaster DNApol‐α180* *D. melanogaster Rcd‐1r*	*D. melanogaster U6:3*	KaramiNejadRanjbar et al. (2018)
*Anopheles gambiae*	*A. gambiae zpg*	*A. gambiae U6*	Kyrou et al. (2018)
*Mus musculus*	*M. musculus vasa* *M. musculus Stra8*	*H. sapiens U6*	Grunwald et al. (2019)

Information is presented in the chronological order of the publications. All are endogenous cis‐regulatory sequences. The genes whose cis‐regulatory sequences were used to drive *Cas9* expression are expressed specifically in the germline. *U6* is a ubiquitously expressed gene encoding a small RNA involved in mRNA splicing. For *Cas9* expression in *M. musculus*, the germline‐specific cis‐regulatory sequences were used in combination with the Cre‐loxP system and constitutive cis‐regulatory sequences

Synthetic regulatory elements containing multiple adjacent binding sites for a small number of transcriptional activators have not been used so far in published gene drive constructs but they might in the future. In general, such synthetic pieces tend to be more universal than endogenous regulatory elements (Schetelig & Wimmer, 2011). For example, the Drosophila‐derived *3xP3* artificial element drives expression in the eyes not only in insects but also in arthropods (Pavlopoulos & Averof, 2005) and planarians (Gonzalez‐Estevez, Momose, Gehring, & Salo, 2003).

The guide RNA gene and the *Cas9* gene can also be activated by enhancers located further away, outside of the original gene drive cassette. Reciprocally, vectors used for gene therapy have been shown to misregulate endogenous genes neighboring the inserted DNA, thereby causing harmful side effects such as leukemia (Browning & Trobridge, 2016). The addition of chromatin insulators into retroviral vectors, to block the activation of nearby genes, seems to be a good solution to this problem, as shown by a recent successful gene therapy (Mamcarz et al., 2019). To our knowledge, none of the published gene drive constructs contain insulators. To limit the activity of gene drive constructs in nontargeted species, we suggest that gene drive vectors should include insulators.

### 2.4 Probability that the CRISPR‐guide RNA recognizes and cuts at a DNA site in the new host (*cut*)

So far, the CRISPR‐Cas9 system has been shown to cut at target sites specified by the guide RNA in all species that have been tested, including animals, plants, bacteria and parasites (Zhang, 2019). We therefore consider here that the probability *cut* that the CRISPR‐guide RNA recognizes and cuts at a DNA site in the new host is simply the probability that the host genome contains a site targeted by the CRISPR‐guide RNA of the gene drive construct. Constructs with multiple guide RNAs have a greater chance for cutting the DNA in a foreign host compared to single‐gRNA constructs. The target site recognized by CRISPR‐Cas9 is 20 nucleotides long followed by a protospacer adjacent motif (PAM), typically NGG (Zhang, 2019). Importantly, studies in yeast and human cells indicate that CRISPR‐Cas9 cleavage activity can still occur with three to five base pair mismatches in the 5′ end (Fu et al., 2013; Hsu et al., 2013; Roggenkamp et al., 2018). As a consequence, we need here to estimate the probability of finding a particular sequence of 17–19 nucleotides (plus one nucleotide that can be either base, located two bases before the 3′ end of the segment) in genomes of interest. The existence of other off‐target cuts with lower sequence similarity to the on‐target site is still under exploration and not entirely understood (Gao, Chuai, Yu, Qu, & Liu, 2019; Zhang, Tee, Wang, Huang, & Yang, 2015). The number of off‐target cuts tends to accumulate with longer and stronger expression of the *Cas9* gene (Kim, Kim, Cho, Kim, & Kim, 2014), suggesting that the range of off‐targets may differ between gene drive cassettes. We consider here only the sequences that are closely related to the on‐target site and therefore provide a lower estimate of the probability *cut*.

A very rough estimate of the probability for a given sequence of 19 nucleotides to be present in a genome of a billion nucleotides can be estimated as follows. Assuming that the 4 nucleotides A, C, G and T are equiprobable, the probability of finding each sequence is 2/4^19^ (the factor 2 stands for the fact that DNA has two strands) which is about 7.10^−12^. In a genome of 3.10^9^ nucleotides such as the human haploid genome, such a sequence should be present with a probability 7.10^−12^ × 3.10^9^, which is approximately 2% (about 30% for a sequence of 17 nucleotides). Note that this is a very crude estimate. It could be higher if one took into account the fact that the DNA contents of AT and GC are different. Also, it does not take into account that an extant DNA sequence is never random. The nonrandom character of genomes is illustrated by the omnipresence of repeated sequences (for example in the human genome the repeats Alu, SINE, LINE etc.) so that if a given sequence is present in a genome, it is likely to be present more than once. Overall it appears that genomes behave as n‐plications of a core set of sequences followed by reduction. This has been observed in yeasts (Escalera‐Fanjul, Quezada, Riego‐Ruiz, & González, 2018), plants (Clark & Donoghue, 2018) and animals (Hermansen, Hvidsten, Sandve, & Liberles, 2016).

The fact that the target sequence is not an arbitrary sequence can increase or decrease the probability according to whether the bias for the sequence of interest and the bias for the sequences in the genome both go in the same direction or not. The probability *cut* would be lower if the existence of repeated sequences was taken into account and if the targeted sequence did not match some of the repeated sequences. By contrast, it would be higher if the sequence matched the “style” of the DNA of a particular organism, that is, its nonrandom content in particular motifs (Fertil et al., 2005). Different mechanisms can lead to the occurrence of repeated sequences in the genome (gene conversion, unequal gene exchanges, transposition, etc.); they have been grouped under the name of “molecular drive” (Dover, 1982). “Molecular drive” is likely to be widespread, as a sizeable proportion of individuals carry local duplications of any sequence of the genome. Furthermore, some conserved repeats may maintain the coexistence of stable rearrangements in some species (Smalec, Heider, Flynn, & O’Neill, 2019), increasing the possibility of unexpected cuts in certain species. All these features may considerably impact the probability of cuts in particular genomes. If the targeted site is present in multiple copies, there is a risk for the gene drive construct to spread across the entire genome. We urge the researchers developing gene drive constructs to make sure that they choose a target site that is very distinct from sequences such as retroelements, LINE, SINE elements that are present in large quantities in genomes (see [Breitwieser, Pertea, Zimin, & Salzberg, 2019] for an updated view of repeats in the human genome).

A recently proposed gene drive that aims at sterilizing mosquito females has the following target site, GTTTAACACAGGTCAAGCGGNGG, which is a highly conserved sequence within the *Doublesex* gene, displaying a 3’ terminal end that contains either a repeated CGG triplet or variant of it. We should note that CGG repeats are quite frequent in genomes (Pan, Man, Roland, & Sagui, 2018; Rabeh, Gaboun, Belkadi, & Filali‐Maltouf, 2018). The 23‐bp sequence is present in 7 species of *Anopheles* mosquitoes, and with one mismatch in at least 6 additional *Anopheles* species (Kyrou et al., 2018). Sequencing of 765 wild‐caught *Anopheles gambiae* mosquitoes identified only one single nucleotide variant within this sequence, and this variant was still permissive to gene drive. Our BLAST searches for this target sequence and for the one used in the published mouse gene drive found fragments of up to 20 bases identical to the 3′ part of the sequence in several genomes, belonging to all three domains of life (Table S1). While a target sequence which accommodates little nucleotide variation such as the *Doublesex* locus can be useful to prevent gene drive resistance, it is then also associated with an increased risk of spread to nontarget species. This trade‐off exists not only for the *Anopheles gambiae* mosquito complex but for any species targeted by a gene drive system.

### 2.5 Probability that the gene drive cassette inserts near the targeted site (*flank*)

The gene drive cassette is designed to bias its transmission by copying itself on the paired chromosome, so that heterozygous individuals carrying initially one copy of the gene drive cassette end up with two copies in their germline cells (Esvelt et al., 2014). This homing process occurs through homology‐directed repair, using homology arms flanking both the gene drive cassette and the guide RNA target site. Therefore, for the gene drive to be active in nontarget species, the DNA containing the *Cas9* gene and the *gRNA* gene should be inserted at the guide RNA target site. *Flank* is the probability that the gene drive cassette lands up at the guide RNA target site.

In case of hybridization, the nontarget species being closely related to the species carrying the initial gene drive, the genomic regions harboring the gene drive cassette are expected to be comparable, so that the probability *flank* should be close to 1.

In case of HT to a distantly related species with no hybridization, the gene drive cassette is likely to insert at a random site in the genome. In this case, *flank* is the probability that it moves from this initial position to a site targeted by its guide RNA (Figure 2). There are several ways such a transposition can happen. Double‐strand DNA breaks, such as the one created at the guide RNA target site by the CRISPR system, are known to induce recombination and various DNA repair mechanisms (Hartlerode & Scully, 2009; Jasin, 1996).

First, the gene drive cassette may move to the guide RNA target site via homology‐directed repair (Figure 2). Such phenomenon has been observed with gene drive constructs that were not inserted initially at the target site but at another position in the genome (Gantz & Bier, 2015; Guichard et al., 2019). The probability of such an event will depend on the length of the flanking homology arms, their percentage of identity, the length of the DNA sequence in between and the position of the donor sequence relative to the cut site (Kanca et al., 2019; Wang, Lee, & Haber, 2017). Unfortunately, as far as we know, no extensive survey of these parameters has been done with respect to homology‐dependent repair using another chromosomal locus as template. A gene drive cassette of 21 kb was found to spread effectively in mosquitoes (Gantz et al., 2015), suggesting that relatively large pieces of DNA can be inserted via homology‐directed repair. In any case, larger inserts tend to show lower efficiency of recombination (Li, Wang, Andersen, Zhou, & Pu, 2014). Gene drive constructs published to date have used flanking sequences of about 1 kb (Gantz & Bier, 2015; Gantz et al., 2015; Grunwald et al., 2019). When linear double‐stranded DNA is used as a template for repair, efficient targeted genome integration can be obtained using flanking sequences that are only 50‐bp long in mammalian cells (Li et al., 2014; Wierson et al., 2018), 100‐bp long in *D. melanogaster* (Kanca et al., 2019) and even 20–40‐bp long in zebrafish (Auer & Del Bene, 2014; Hisano et al., 2015; Wierson et al., 2018), *Xenopus laevis* (Nakade et al., 2014), *Bombyx mori* (Nakade et al., 2014) or the nematode *C. elegans* (Paix et al., 2014). Whether such short sequences could also favor transposition of the gene drive cassette to the guide RNA target site remains unknown. If repeat sequences exist both near the initial insertion site of the gene drive and the guide RNA site, they may facilitate such transposition (Figure 2). Evaluating the parameter *flank* thus requires an assessment of the distribution of repeats in potential nontarget species.

As emphasized by Salzberg and coworkers, the human genome sequence, while far more complete than most animal genomes, is still made of 473 scaffolds and comprises 875 gaps (Breitwieser et al., 2019). As expected, the gaps encompass regions with a variety of repeats, some of them still poorly characterized, in particular in centromeric and pericentromeric regions. This precludes accurate analysis of their distribution, and the situation is even worse for other genomes. For instance, the transposon‐derived Alu repeats (~300 bp long), that are present in primate genomes at more than one million copies, are widely variable, within and among chromosomes (Grover, Mukerji, Bhatnagar, Kannan, & Brahmachari, 2004). Interestingly this distribution is biased toward proximity of protein‐coding gene regulatory regions (Lavi & Carmel, 2018). In addition to our somewhat limited knowledge of the distribution of repeats in mammalian genomes, we have to consider that when a species is represented by a large number of individuals there are many copy number variants, in particular in repeated regions (Monlong et al., 2018).

Second, the gene drive cassette may move to the guide RNA target site via a TE. It is well established that TEs play a considerable role in displacement of genes or regulatory regions across genomes (Chen & Yang, 2017). As a matter of fact, TEs are recognized as a frequent cause of genetic diseases (see, e.g., [Larsen, Hunnicutt, Larsen, Yoder, & Saunders, 2018; Song et al., 2018] and references therein).

The events discussed above may seem extremely rare, but they do not have to occur right after the gene drive inserted in a genome. The gene drive cassette may stay dormant for a few generations, and there can be many trials and errors in various individuals before an insertion occurs at the guide RNA target site. Of note, gene drive constructs containing several guide RNAs (Esvelt et al., 2014) increase the chance that the drive moves to a targeted site.

### 2.6 Probability that the immune system does not eliminate Cas9‐expressing cells (*immune*)

The Cas9 protein is derived from the bacteria *Streptococcus pyogenes* (Zhang, 2019) and can trigger an immune response in mice (Chew, 2018; Chew et al., 2016). During the gene drive multiplication process, germline cells produce Cas9 proteins to cut the DNA and insert the gene drive cassette (Figure 2). These germline cells may thus present Cas9 fragments on their surface.

In insects there is no adaptive immune system (Lemaitre & Hoffmann, 2007), so the presence of Cas9 is unlikely to trigger an immune response. However, double‐stranded RNAs larger than 30 bp can be recognized by Dicer2 and activate the RNA interference pathway, leading to their degradation (Elbashir, Lendeckel, & Tuschl, 2001; Gammon & Mello, 2015). Guide RNAs are about 100 nucleotide long including the target site and they contain hairpins shorter than 15 bp (Bassett & Liu, 2014), so they should not be recognized by Dicer2. In summary, present knowledge suggests that gene drives would not be hampered by the immune system in insects.

In vertebrates, if Cas9 proteins accumulate in somatic tissues at a late stage during development due to leakage of the cis‐regulatory regions controlling *Cas9* gene expression, Cas9 fragments may be recognized as foreign molecules and trigger an adaptive immune T cell response, leading to the potential elimination of the Cas9‐expressing cells and a probable decrease in fitness of the individual carrying the gene drive (Chew, 2018). The expression of *Cas9* at an early stage of development may also activate an immune response if the gene drive carrier has inherited anti‐Cas9 antibodies from its mother, as maternal immunoglobulins G have been shown to cross the placental barrier and the intestine, and to be maintained for a long time in the fetus after birth (Madani & Heiner, 1989; Roopenian & Akilesh, 2007). However, testes—and maybe ovaries—appear to readily accept foreign antigens without the induction of an immune response in several mammals (Li, Wang, & Han, 2012; Mellor & Munn, 2008; Simpson, 2006), so that germline cells expressing *Cas9* may not be eliminated by the immune system. Furthermore, guide RNAs produced from gene drive constructs are not expected to elicit an immune response in vertebrates, as they do not carry 5’‐triphosphate ends (Kim et al., 2018; Wienert, Shin, Zelin, Pestal, & Corn, 2018). Clearly, our knowledge in immunology is presently too sparse to anticipate how gene drive systems will interact with the immune system in vertebrates.

*S. pyogenes* is a facultative pathogenic bacteria mostly restricted to humans, with about 10%–20% of the population being asymptomatic carriers (Roberts et al., 2012; Shaikh, Leonard, & Martin, 2010). It is thus no surprise that recent investigations have found anti‐Cas9 antibodies and Cas9‐reactive T cells in several human populations (Charlesworth et al., 2019; Wagner et al., 2019). It is thus possible that certain humans are immune to gene drives, and future studies will undoubtedly shed light on this question. The presence of *S. pyogenes* has also been documented in a few animals such as macaques, mice, dogs, hedgehogs, rabbits and sheep ([Chen et al., 2019; Vela et al., 2017] and references therein). Whether these vertebrates may also be immune to gene drives remains to be investigated.

So far, all published gene drive constructs have used Cas9 from *S. pyogenes* (SpCas9). Other Cas proteins with activity similar to SpCas9 are available (Zhang, 2019) and could potentially be used for gene drive technology. As they are derived from other types of bacteria, their immunogenicity and their associated probability *immune* would have to be specifically assessed.

### 2.7 Probability of nonextinction of the drive (*nonextinct*)

Once the drive successfully introduced into the genome, its fate will depend on its ability to distort its segregation, on chance, on its associated selective value and on the probability that the nontarget population evolve resistance to the gene drive. Several stochastic and deterministic models have been composed to assess the dynamics of gene drive alleles once they are introduced in substantial amount in a targeted population, taking into account the possible costs of harboring a gene drive as well as the appearance of resistance to gene drive (Deredec et al., 2008; Marshall, 2009; Unckless, Messer, Connallon, & Clark, 2015).

For simplicity, we treat here the probability that a drive, initially present in a single individual or so, is not eliminated immediately by mere chance due to sampling effect and reach significant numbers of individuals in the nontarget population. Even if an allele manages to be present in more than one half of the gametes from heterozygous individuals, it can still disappear rapidly due to random processes. If not, then it can invade the population. This process has been modeled as a branching process by Bienaymé (1845), actually published by Cournot (1847), and Watson and Galton (1875), see (Bru, 1991; Kendall, 1975). The probability of extinction, starting with one replicator, is the lowest root of equation $G\left(x\right)=x$, where $G\left(x\right)$ is the generating function of the law describing the number of copies left per generation by one replicator. Here, it is relevant to assume the distribution to follow a Poisson law. If the population as a whole is stable, each “normal” gene leaves on average one copy of itself in the next generation, that is, on average 1/2 through male gametes and 1/2 through female ones. The drive will then leave a number of copies of itself equal to the sum of the proportions which it represents in male (*λ*_m_) and in female (*λ*_f_) gametes: $\lambda =\lambda_{m}+\lambda_{f}$. Typically, λm and $\lambda_{f}$ are around 0.7–0.9 for gene drives tested in mice and insects (Gantz & Bier, 2015; Gantz et al., 2015; Grunwald et al., 2019; Hammond et al., 2016; KaramiNejadRanjbar et al., 2018; Kyrou et al., 2018) so that a reasonable estimate of λ is 1.4–1.8. According to the “Bienaymé, Galton, Watson” model, the extinction probability for a Poisson law of parameter λ is such that $G\left(x\right)=e^{\lambda \left(x-1\right)}x$. With λ=1.4 (resp. 1.8), it gives x≃0.5 (resp. 0.27) and the probability of invasion (1 − *x*) is thus around 0.5–0.7.

For GM plants methods allowing to reduce the risk of contamination of non‐GM plants have been proposed (see above). However, no experimental situation has yet allowed to test the efficiency of such measures. Moreover, despite a strong concern about these questions from citizens and biosafety agencies, none of the methods which could reduce contamination has been developed in commercialized varieties.

A mathematically elegant solution aimed at preventing a drive from invading a nontarget species has been proposed (Barton & Turelli, 2011; Tanaka, Stone, & Nelson, 2017). It is inspired from the population genetics of hybrid zones where a gene can invade only if it reaches a sufficiently high frequency locally. Such threshold‐dependent gene drives appear as a good solution to prevent contamination of nontarget populations. Calculations show that the selective disadvantage of such drives should be more than 0.5 to invade the target population and less than 0.697 so that it cannot invade nontarget populations when starting from a low initial frequency. Note that these values hold only when the segregation distortion of the drive is 100% and that the range is narrower if segregation distortion is lower. One can hope that any drive released in nature will display these characteristics (100% efficiency conversion and 0.5 < *s* < 0.697). However, given the narrow range of values for *s*, it will not be easy to achieve such drives, especially when gene drive individuals are released in diverse places in nature where various ecological factors can affect fitness values.

## 3 DISCUSSION

Based on our examination of the seven parameters of the DRAQUE equation (Table 3), the probability that a gene drive transfers to another species can have values ranging from 0 to 0.5 per year in the worst‐case scenario (one hybridization event occurring per year, the guide RNA site is present in the nontarget genome, the nontarget species has high levels of homologous recombination). Our current estimate of the overall risk (Table 3) remains nevertheless very crude and asks for further studies to refine this estimate.

**TABLE 3:** Overview of the various parameters of the DRAQUE equation

Parameter	Rough estimate
*transf*	0.035 transfer events or more to at least one species per thousand years for a given transposable element; may be lower for a gene drive construct
*hyb*	unclear, such events are possible in species closely related to the target species
*express*	close to 1 in vertebrate species for gene drives targeting mice, close to 1 in Diptera species for gene drives targeting mosquitoes or flies
*cut*	0.02 in a random genome of human size
*flank*	unclear
*immune*	close to 1 for nontarget insect species, may be lower for nontarget vertebrate species
*nonextinct*	0.5–0.7

We did not treat here the risk of contamination of another population, within the targeted species, but the same formula could be used in principle for this case. Furthermore, our equation does not take into account the phenotypic effect of the drive on the contaminated species. A drive may display no phenotypic effects in the nontarget species that it invaded. If the gene drive was designed to eliminate a target population, then it is more likely to eliminate the nontarget species. The drive may also have unexpected effects, for example the creation of nontarget mutations in somatic cells due to perdurance of Cas9 expression (Guichard et al., 2019).

To prevent contamination, several containment strategies for laboratory experiments have been proposed (Akbari et al., 2015; Benedict et al., 2008; National Academies of Sciences & Medicine, 2016) as well as gene drives split in two different constructs (Benedict et al., 2008; DiCarlo et al., 2015) and synthetic target sites (Champer et al., 2019). Based on our survey of the various parameters, we suggest further design strategies to minimize the risk of transfer to a nontarget species: the addition of insulators (see above), and the choice of a guide RNA target site that is not close to repeated sequences or to the centromere, to avoid rearrangements and increased probability of creating an active gene drive in a nontarget species.

Here, we treated probabilities for a standard gene drive construct, but the risk should be estimated for each particular gene drive construct. New types of self‐limiting gene drives have been proposed in recent years to try to limit the spread of gene drives spatially or temporally: toxin‐antidote systems including Medea (Buchman, Marshall, Ostrovski, Yang, & Akbari, 2018), CleaveR (Oberhofer, Ivy, & Hay, 2019), Killer‐Rescue (K‐R) (Gould, Huang, Legros, & Lloyd, 2008; Webster, Vella, & Scott, 2019), one or two‐locus underdominance (Dhole, Vella, Lloyd, & Gould, 2018) and Daisy‐Chain drives (Noble et al., 2019). The DRAQUE parameters would have to be evaluated specifically for these particular cases.

We have restricted our evaluation of the probability of accidental spread of gene drive constructs to genomes considered as fairly stable entities. However, genomes are dynamic structures. The process of gene duplication (or even n‐plication) is commonplace (Clark & Donoghue, 2018; Harari, Ram, & Kupiec, 2018; Moriyama & Koshiba‐Takeuchi, 2018). Local amplification of sequences is also very frequent (Liu et al., 2019; Traynor et al., 2019) and this may increase the probability of accidents to a further unknown level.

The DRAQUE formula does not cover all the risks associated with gene drive. In addition to the risk of transfer to another species, gene drive designed to eliminate a target population may have additional ecological consequences that are not treated here.

Living with highly evolved technologies entails high risks for individuals and societies. Here, we have attempted to evaluate circumstances where risks could be identified, but this assumes that we are aware of all the natural processes coupled with the technologies of interest. Besides the technology itself—which can be properly monitored and steered—there is an additional risk that is seldom taken into account, the risk derived from the way the organizations that implement the technologies manage them (Perrow, 2011). We have not tackled this question here, but it is an obvious place of much concern. The way novel biological technologies have been used recently—see modification of human babies with deletion of a surface cell receptor (Sand, Bredenoord, & Jongsma, 2019)—should remind us that rogue or reckless scientists may use gene drive approaches without proper risk assessment.

Overall, our study reveals that there is a need for more detailed investigations of the different factors influencing the probability of a gene drive contaminating another species before any release in the wild population is ever considered. We hope that our paper will trigger discussions and progress in the ethics of gene drive technology.

## Supporting information

Table S1

## Data Availability

This article does not contain data.
