# Supplementary material for: Evaluating the probability of CRISPR‐based gene drive contaminating another species
Source: Evol Appl. 2020 Apr 17;13(8):1888–905. doi: 10.1111/eva.12939 (PMC7463340; doi:10.1111/eva.12939)
Supplement: Supplementary file 1 — Table S1 [file EVA-13-1888-s001.pdf]

Table S1. Results of BLAST searches for target sequences of two gene drive constructs published previously (Kyrou et al. 2018; Grunwald *et al.* 2019).

The BLAST search was performed using 20-nucleotide-long queries on the nr database with default parameters on https://blast.ncbi.nlm.nih.gov/Blast.cgi?PROGRAM=blastn&PAGE_TYPE=BlastSearch&LINK_LOC=blasthome. Note that the BLAST algorithm does not retrieve results when the query sequence contains an N at the antepenultimate position. In consequence, the “N” in “NGG” was replaced by each of the four nucleotide letters and four BLAST searches were performed for each target sequence. We examined only the first 100 hit sequences and selected here the ones with at least 16/20 nucleotide sites identical to the query and harbouring the protospacer adjacent motif (PAM) NGG. Hit sequences are shown by taxonomic group for each query sequence. Note that sequences are not always deposited in the nr database (for example the *Anopheles* sequences were not retrieved by our BLAST searches), so that this table does not reflect the full extent of all published sequence data. Note also that the target sequence for the tyrosinase locus is incorrectly labelled in Extended Data Figure 2 of Grunwald *et al.* 2019 (Grunwald *et al.* 2020).

Target: *doublesex* in 6 *Anopheles* species			
TAACACAGGTCAAGCGG**TGG**	TAACACAGGTCAAGCGG**AGG**	TAACACAGGTCAAGCGG**CGG**	TAACACAGGTCAAGCGG**GGG**
**Insects**	**Insects**	**Insects**	**Insects**
Anopheles stephensi	*Leptidea sinapis*		*Spodoptera litura*
			Habropoda laboriosa
**Fungi**	**Fungi**	**Fungi**	**Fungi**
Aspergillus sojae		*Uncinocarpus reesii*	
Aspergillus nomius			
Aspergillus flavus			
Aspergillus oryzae			
Podospora comata			
Podospora anserina			
Fonsecaea monophora			
Fonsecaea nubica			
**Apicomplexans**	**Apicomplexans**	**Apicomplexans**	**Apicomplexans**
	Plasmodium cynomolgi		*Plasmodium coatneyi*
	Plasmodium vivax		
**Bacteria**	**Bacteria**	**Bacteria**	**Bacteria**
Aerococcus urinaehominis	*Desulfitobacterium hafniense*	*Pseudarthrobacter sp*	*Enterococcus gallinarum*
Plantomycetes sp. varia	*Vampirococcus sp.*	*Altererythrobacter sp.*	*Enterococcus sp.*
Marinobacter sp	*Bacillus licheniformis*	*Streptomyces sp.*	*Serratia sp.*
Thermaerobacter marianensis		*Nitrospira sp.*	*Enterococcus casseliflavus*
		Alistipes sp.	*Streptomyces venezuelae*
		Sporosarcina sp.	*Desulfovibrio vulgaris*
		Sphingomonas paucimobilis	*Micromonospora auratinigra*
		Sodalis glossinidius (insect symbiont)	*Streptomyces venezuelae*
**Birds**	**Birds**	**Birds**	**Birds**
Anser cygnoides domesticus			

Streptopelia turtur			
**Mammals**	**Mammals**	**Mammals**	**Mammals**
Homo sapiens		*Peromyscus leucopus*	*Homo sapiens*
		Rhinopithecus bieti	*Pan troglodytes*
		Rhinopithecus roxellana	*Pongo abelii*
			Canis lupus (dingo and familiaris)
**Bony fishes**	**Bony fishes**	**Bony fishes**	**Bony fishes**
Chanos chanos	*Chanos chanos*	*Myripristis murdjan*	*Takifugu rubripes*
Salarias fasciatus	*Salmo trutta*	*Denticeps clupeoides*	*Amphiprion ocellaris*
Parambassis ranga	*Anabas testudineus*		*Acanthochromis polyacanthus*
Betta splendens	*Astatotilapia calliptera*		*Haplochromis burtoni*
	Oreochromis niloticus		*Oreochromis niloticus*
	Scophthalmus maximus		*Stegastes partitus*
	Maylandia zebra		*Maylandia zebra*
			Neolamprologus brichardi
			Pundamilia nyererei
			Gasterosteus aculeatus
			Scleropages formosus
			Lateolabrax maculatus
**Lancelets**	**Collembolas**	**Molluscs**	**Lancelets**
Branchiostoma floridae	*Folsomia candida*	*Crassostrea gigas*	*Branchiostoma floridae*
**Plants**	**Plants**	**Plants**	**Plants**
Prunus dulcis	*Ipomoea trifida*	*Oryza sativa*	
Citrus sinensis		*Solanum lycopersicum*	

Target: *tyrosinase* in *Mus musculus*			
ATGGCCGATAGGTGCAT**AGG**	ATGGCCGATAGGTGCAT**CGG**	ATGGCCGATAGGTGCAT**GGG**	ATGGCCGATAGGTGCAT**TGG**
**Mammals**	**Mammals**	**Mammals**	**Mammals**
		Pan troglodytes	*Cricetulus griseus*
			Grammomys surdaster
			Homo sapiens
	Amphibians	**Birds**	*Mus caroli*
	Rhinatrema bivittatum	*Streptopelia turtur*	*Mus musculus*
			Mus pahari
			Peromyscus leucopus
**Bony fishes**	**Bony fishes**	**Bony fishes**	
Gadus morhua	*Sparus aurata*	*Anabas testudineus*	
Betta splendens	*Sphaeramia orbicularis*	Cottoperca gobio	
		Gadus morhua	
		Parambassis ranga	
**Insects**	**Insects**	**Insects**	**Insects**

		Drosophila elegans	*Diabrotica virgifera*
			**Collembola**
			Folsomia candida
**Fungi**	**Fungi**	**Fungi**	**Fungi**
			Cercospora sojina
			Vonarxia vagans
**Apicomplexans**	**Euglenozoans**	**Apicomplexans**	**Apicomplexans**
	Trypanosoma rangeli		
	**Plants**		
	Physcomitrella patens		
**Bacteria**	**Bacteria**	**Bacteria**	**Bacteria**
Pseudomonas monteilii	*Arthrobacter sp.*	*Mycobacterium intracellulare*	*Candidatus Planktophila dulcis*
Pseudomonas putida	*Moorella thermoacetica*	*Pseudomonas arsenicoxydans*	*Rhodothermus marinus*
Streptomyces autolyticus	*Pseudomonas lurida*		Uncultured bacterium
Streptomyces malaysiensis	*Pseudomonas sp.*		
Streptomyces sp. M56			
Vibrio coralliilyticus			
Vibrio tubiashii			
	Marine virus		

References

Grunwald, H. A., Gantz, V. M., Poplawski, G., Xu, X. R. S., Bier, E., & Cooper, K. L. (2019). Super-Mendelian inheritance mediated by CRISPR–Cas9 in the female mouse germline. *Nature*, *566*(7742), 105-109. https://doi.org/10.1038/s41586-019-0875-2

Grunwald, H. A., Gantz, V. M., Poplawski, G., Xu, X. R. S., Bier, E., & Cooper, K. L. (2020). Author Correction: Super-Mendelian inheritance mediated by CRISPR–Cas9 in the female mouse germline. *Nature 577*, E8. https://doi.org/10.1038/s41586-019-1861-4

Kyrou, K., Hammond, A. M., Galizi, R., Kranjc, N., Burt, A., Beaghton, A. K., ... & Crisanti, A. (2018). A CRISPR–Cas9 gene drive targeting doublesex causes complete population suppression in caged Anopheles gambiae mosquitoes. *Nature biotechnology*, *36*(11), 1062-1066. https://doi.org/10.1038/nbt.4245
